# Correlations between the Memory-Related Behavior and the Level of Oxidative Stress Biomarkers in the Mice Brain, Provoked by an Acute Administration of CB Receptor Ligands

**DOI:** 10.1155/2016/9815092

**Published:** 2015-12-29

**Authors:** Marta Kruk-Slomka, Anna Boguszewska-Czubara, Tomasz Slomka, Barbara Budzynska, Grazyna Biala

**Affiliations:** ^1^Department of Pharmacology and Pharmacodynamics, Medical University of Lublin, Chodzki 4a Street, 20-093 Lublin, Poland; ^2^Department of Medical Chemistry, Medical University of Lublin, Chodzki 4a Street, 20-093 Lublin, Poland; ^3^Department of Mathematics and Medical Biostatistics, Medical University of Lublin, Jaczewskiego 4 Street, 20-954 Lublin, Poland

## Abstract

The endocannabinoid system, through cannabinoid (CB) receptors, is involved in memory-related responses, as well as in processes that may affect cognition, like oxidative stress processes. The purpose of the experiments was to investigate the impact of CB1 and CB2 receptor ligands on the long-term memory stages in male Swiss mice, using the passive avoidance (PA) test, as well as the influence of these compounds on the level of oxidative stress biomarkers in the mice brain. A single injection of a selective CB1 receptor antagonist, AM 251, improved long-term memory acquisition and consolidation in the PA test in mice, while a mixed CB1/CB2 receptor agonist WIN 55,212-2 impaired both stages of cognition. Additionally, JWH 133, a selective CB2 receptor agonist, and AM 630, a competitive CB2 receptor antagonist, significantly improved memory. Additionally, an acute administration of the highest used doses of JWH 133, WIN 55,212-2, and AM 630, but not AM 251, increased total antioxidant capacity (TAC) in the brain. In turn, the processes of lipids peroxidation, expressed as the concentration of malondialdehyde (MDA), were more advanced in case of AM 251. Thus, some changes in the PA performance may be connected with the level of oxidative stress in the brain.

## 1. Introduction

It has been widely reported that intense oxidative stress-related processes in the brain are one of the main causal factors involved in the impairment in cognitive functions through two critical changes in the brain. First, a decrease in neurotransmitters, essential for memory and learning functions, for example, acetylcholine (ACh), as well as a decrease in level of natural antioxidants in the brain by activating microglia, a source of reactive oxygen species (ROS), has been reported [1, 2]. The formation of ROS and other free radicals during metabolism is an important and normal process that is ideally compensated by an elaborate endogenous antioxidant system. However, excessive radical production and their accumulation result in oxidative stress, which has been implicated in mechanisms responsible for oxidative injury of neurons by causing damage of cell structures, including lipids, membranes, and proteins [1]. The central nervous system (CNS) is very susceptible to oxidative stress. Additionally, it contains large amounts of free-radical generating iron and substances like ascorbate, glutamate, and unsaturated fatty acids that easily undergo redox-reaction leading to radical formation [3]. Peroxidation of lipids, which are abundant constituent of neurilemma, can directly destroy the structural integrity of membranes and lead to significant changes in their biophysical functions. Moreover, malondialdehyde (MDA), the product of lipid peroxidation, is a neuronal toxin and may impair protein function [4].

Additionally, ROS are highly neurotoxic and thereby induce oxidative damage connecting with many neurodegenerative disorders, for example, Alzheimer disease (AD) [5–7]. Imbalances between local ROS and antioxidant capacity, neuroinflammation, and accumulation of oxidatively modified proteins within the brain potentiate neurodegeneration and impair cognitive function causing memory deficits. Additionally, free radicals trigger neuroinflammation by upregulated production of proinflammatory factors, such as cytokines and chemokines. These factors, especially tumor necrosis factor-*α* (TNF-*α*) and interleukin-1*β* (IL-1*β*), can induce chronic inflammation that causes the loss of synapses, neuronal death, and consequently cognitive dysfunction characteristic for AD [8]. For instance, higher concentrations of oxidative damage to protein, nucleic acids, and lipids, as well as lower activities of natural antioxidants, were observed in patients with AD [9].

There is no effective treatment available for human disturbances associated with memory impairment. However, there is intense research into developing new treatments for cognitive decline, with some focusing on searching for compounds to the more conventional pharmacological targets. Many possible pharmacological strategies are based on the fact that oxidative stress can result in cognitive impairments; thus the drugs that are able to inhibit oxidative processes (antioxidants) seem to be very useful for the treatment of memory deficits [10–14].

One of the promising strategies for the treatment of cognitive impairments is connected with endocannabinoid system, including cannabinoid (CB) receptors [15, 16]. Currently, two types of CB receptors are known: CB1 and CB2. The first ones were found in the brain, especially in the basal ganglia, amygdala and cerebellum, and peripheral tissues. CB1 receptors are also highly expressed in basolateral amygdala (BLA), the medial prefrontal cortex (mPFC) and the hippocampus, and the main brain regions involved in emotional-related responses, for example, cognitive processes [17]. Because activation of CB1 receptors regulates the release of neurotransmitters which are involved in excitotoxic neurodegenerative processes, CB1 receptor ligands can protect against excitotoxicity and promote neurogenesis. However, neuroprotective properties of cannabinoids can be also independent of the presence of the CB1 receptors [17, 18]. In turn, CB2 receptors are located predominantly, but not exclusively, in the periphery on immunological tissues; however, recent study revealed that these receptors are also located on the brain areas, such as cerebellum and hippocampus. Moreover, CB2 receptors have been identified in microglial cells [19, 20]. Stimulation of the CB2 receptor attenuates oxidative stress processes and reduces neuroinflammation by suppression of microglial activation and controls the production of inflammatory mediators. Thus, selective CB2 receptor ligands may reduce neuroinflammatory processes and enhance neurogenesis [21].

However, biochemical and behavioral effects of cannabinoids, especially of CB2 receptor ligands, are more complex.

Our interests have been focused on the neurobiological mechanisms of endocannabinoid system in the context of the memory-related processes associated with the level of oxidative stress in the brain. To better understand the involvement of this system in the memory-related responses, we examined the influence of selective or nonselective CB receptor ligands on the long-term memory acquisition and consolidation in mice using the passive avoidance (PA) test. PA task is used to test the effect of novel compounds on the memory as well as to study the complex mechanisms involved in memory and learning processes. In this test, animals learn to avoid an environment in which an aversive stimulus was previously delivered.

Additionally, we would like to improve knowledge on the biochemical-related effect of endocannabinoid system in the context of occurrence of oxidative stress. In our study, the level of oxidative stress was assessed by determination of total antioxidant capacity (TAC), activity of superoxide dismutase (SOD), an antioxidant enzyme responsible for inactivation of superoxide anion radical O_2_^•−^, and concentration of malondialdehyde (MDA), the biomarker of lipids peroxidation processes, in the brain of mice after an acute administration of selective or nonselective CB receptor ligands.

Finally, correlation analysis was performed to determine how and whether changes in PA performance are associated with changes in the concentration of oxidative stress biomarkers in the brain of mice.

Our results are discussed in the context of the involvement of endocannabinoid system in cognition- and oxidative stress-related processes. CB receptor ligands, due to their extensive pharmacological and biochemical properties, could become a new alternative for the prevention or treatment of human memory disorders associated with oxidative stress in the brain.

## 2. Materials and Methods

### 2.1. Animals

The experiments were carried out on naive male Swiss mice (Farm of Laboratory Animals, Warszawa, Poland) weighing 20–30 g. The animals were maintained under standard laboratory conditions (12 h light/dark cycle, room temperature 21 ± 1°C) with free access to tap water and laboratory chow (Agropol, Motycz, Poland) in their home cages and adapted to the laboratory conditions for at least one week. Each experimental group consisted of 8–12 animals. All behavioral experiments were performed between 8:00 and 15:00 and were conducted according to the National Institute of Health Guidelines for the Care and Use of Laboratory Animals and to the European Community Council Directive for the Care and Use of Laboratory Animals of September 22, 2010 (2010/63/EU), and approved by the local ethics committee.

### 2.2. Drugs

The CB compounds tested were the following:

 
*WIN 55,212-2* (0.25, 0.5, and 1.0 mg/kg) (Tocris, USA), a mixed CB1/CB2 receptor agonist,
 
*AM 251* (0.25, 0.5, 1.0, and 3.0 mg/kg) (Tocris, USA), a selective CB1 receptor antagonist,
 
*JWH 133* (0.25, 0.5, 1.0, and 2.0 mg/kg) (Tocris, USA), a potent selective CB2 receptor agonist,
 
*AM 630* (0.25, 0.5, 1.0, 2.0, and 3.0 mg/kg) (Tocris, USA), a competitive CB2 receptor antagonist.

All CB compounds were suspended in a 1% solution of Tween 80 (Sigma, St. Louis, MO, USA) in saline solution (0.9% NaCl) and administered intraperitoneally (ip) at a volume of 10 mL/kg. Fresh drug solutions were prepared on each day of experimentation. Control groups received saline with Tween 80 injections at the same volume (vehicle) and by the same route of administration.

### 2.3. Behavioral Effects of CB Compounds

Experimental doses of CB receptor ligands used for behavioral experiments and procedures were chosen accordingly to those frequently used in literature [22–29].

#### 2.3.1. Locomotor Activity

*(1) Experimental Procedure.* Locomotion of mice was recorded individually in round actometer cages (Multiserv, Lublin, Poland; 32 cm in diameter, two light beams) kept in a sound-attenuated experimental room. Two photocell beams, located across the axis, automatically measured animal's movements.

*(2) Treatment.* Horizontal locomotor activity was measured immediately after injection of selective or nonselective CB receptor ligands: WIN 55,212-2 (0.25, 0.5, and 1.0 mg/kg, ip); AM 251 (0.25, 0.5, 1.0, and 3.0 mg/kg, ip); JWH 133 (0.25, 0.5, 1.0, and 2.0 mg/kg, ip); AM 630 (0.25, 0.5, 1.0, 2.0, and 3.0 mg/kg, ip) or vehicle for the control group. Locomotor activity, that is, the number of photocell beam breaks, was automatically recorded for 60 min.

#### 2.3.2. Memory-Related Responses

*(1) Experimental Procedure.* Memory-related responses were measured by the passive avoidance (PA) test. According to Venault et al. [28] the step-through passive avoidance task may be recognized as a measure of short- and long-term memory. In our experiments we used the procedure of PA task, which is commonly approved in the assessment of memory-related responses [22, 30–33] and described in detail in our previous articles [11, 34].

The apparatus of PA consisted of two-compartment acrylic box with a lighted compartment (10 × 13 × 15 cm) and darkened compartment (25 × 20 × 15 cm). The light chamber was illuminated by a fluorescent light (8 W) and was connected to the dark chamber which was equipped with an electric grid floor. Entrance of the animals to the dark box was punished by an electric foot shock (0.2 mA for 2 s).

Depending on the procedure used, PA test allows examining different durations of memory (short-term and long-term memory) according to the period between training and test, as well as different stages of memory (acquisition or consolidation) according to the time of drug treatment.

On the first day of training (pretest), mice were placed individually into the light compartment and allowed to explore the light box. After 30 s, the guillotine door was raised to allow the mice to enter the dark compartment. When the mice entered the dark compartment, the guillotine door was closed and an electric foot shock (0.2 mA) of 2 s duration was delivered immediately to the animal via grid floor. The latency time for entering the dark compartment was recorded (TL1). If the mouse failed to enter the dark box within 300 s, it was placed into this dark box, the door was closed, and electric foot shock was delivered to the animal. In this case, TL1 value was recorded as 300 s.

In the subsequent trial (test, retention), 24 h later for the long-term memory, the same mice were again placed individually in the light compartment of the PA apparatus. After a 30 s adaptation period in the light (safe) chamber, the door between the compartments was raised and the time taken to reenter the dark compartment was recorded (TL2). No foot shock was applied in this trial. If the animal did not enter the dark compartment within 300 s, the test was stopped and TL2 was recorded as 300 s.

Pretraining (before the first trial, before pretest) drug administration should interfere with the acquisition of information, while the immediate posttraining drug administration (after the first trial, after pretest) should exert an effect on the process of consolidation. This kind of procedure is commonly approved in the assessment of memory-related responses in variety of pharmacological animal models of memory [22, 33].

*(2) Treatment.* The first step of experiment was designed to evaluate the influence of CB compounds on the acquisition of long-term memory. For this purpose, CB receptor ligands, WIN 55,212-2 (0.25, 0.5, and 1.0 mg/kg, ip); AM 251 (0.25, 0.5, 1.0, and 3.0 mg/kg, ip); JWH 133 (0.25, 0.5, 1.0, and 2.0 mg/kg, ip); AM 630 (0.25, 0.5, 1.0, 2.0, and 3.0 mg/kg, ip), or saline was administered 30 min before the first trial (pretraining) and mice were retested 24 h later.

The second set of experiments was designed to investigate the effects of CB compounds on the consolidation of long-term memory. For this purpose, the independent groups of mice received injections of CB receptor ligands: WIN 55,212-2 (0.25, 0.5, and 1.0 mg/kg, ip); AM 251 (0.25, 0.5, 1.0, and 3.0 mg/kg, ip); JWH 133 (0.25, 0.5, 1.0, and 2.0 mg/kg, ip); AM 630 (0.25, 0.5, 1.0, 2.0, and 3.0 mg/kg, ip) or saline immediately after the first trial (posttraining) and the mice were retested 24 h later.

### 2.4. Biochemical Effects of CB Compounds

Experimental doses of CB receptor ligands used for biochemical experiments were chosen according to results obtained in our presented behavioral experiments above.

#### 2.4.1. Collection of Tissues

Following the PA test, all mice that were administered with the CB compounds were anesthetized and decapitated and the whole brain was carefully taken out and rinsed in isotonic saline to remove blood. Then the tissues were homogenized in 10 volumes of 20 mM TRIS-HCl buffer (pH 7.4) on ice for 20 s and centrifuged at 12000 g for 30 min at 4°C to obtain supernatants, which were used for further study. Total antioxidant capacity (TAC), activity of superoxide dismutase (SOD), and concentration of malondialdehyde (MDA) were determined in such prepared supernatants spectrophotometrically with use of EPOCH microplate reader and HITACHI 2800 apparatus.

#### 2.4.2. Biochemical Estimations

*Determination of Protein Content*. The protein content was determined by the Bradford method [35] using BSA as the standard.

*Determination of TAC by Ferric Reducing Ability of Plasma (FRAP) Assay*. The tissue antioxidant ability was carried out on brain homogenates according to the modified method of Benzie and Strain [36] adapted to specific tissue and microplate assays. The method is based on evaluation of antioxidant capacity of a tissue by estimating the concentration of all substances able to reduce ferric ions. The course of reaction of Fe(III)-tripyridyltriazine (Fe(III)-TPTZ) reduction to blue Fe(II)-tripyridyltriazine (Fe(II)-TPTZ) is determined spectrophotometrically at 573 nm. 

*Determination of SOD Activity*. The activity of SOD was measured with use of ready-to-use diagnostic kits RANSOD by Randox. The method employs xanthine and xanthine oxidase (XOD) to generate superoxide radicals which react with nitroblue tetrazolium to form red formazan dye. The superoxide dismutase activity is then measured by the degree of inhibition of the reaction. The increase in absorbance at 505 nm is read. 

*Estimation of MDA Concentrations*. MDA was measured by the thiobarbituric acid (TBA) reaction [37]. Briefly, 0.5 mL of tissue homogenate supernatant was mixed with 2.5 mL 1.22 M TCA in 0.6 M HCl and allowed to stand for 15 min. Then 1.5 mL of 0.9% TBA was added and the mixture was incubated for 30 min in a boiling water bath. After cooling 4 mL of n-butanol was added and the mixture was shaken vigorously. The samples were centrifuged at 1500 g for 10 min and then the absorbance of organic phase was measured at 532 nm with respect to blank (n-butanol alone). The concentration of MDA was read from the standard curve obtained by using malondialdehyde bis-dimethyl acetal.

### 2.5. Statistical Analysis

#### 2.5.1. Behavioral and Biochemical Experiments

The data were expressed as the means ± standard error of the mean (SEM). The statistical analyses were performed by the one-way analysis of variance (ANOVA). One-way ANOVA with Tukey's posttest was performed using GraphPad Prism version 5.00 for Windows, GraphPad Software, San Diego, California, USA, http://www.graphpad.com/. The confidence limit of *p* < 0.05 was considered statistically significant.

For the locomotor activity, the total number of photocell beam breaks was measured.

For the memory-related behaviors, the changes in PA performance were expressed as the difference between retention and training latencies and were taken as the latency index (LI).

LI was calculated for each animal and reported as the ratio:

 LI = TL2 − TL1/TL1,
 TL1, the time taken to enter the dark compartment during the training,
 TL2, the time taken to enter the dark compartment during the retention [11, 22, 31, 33, 34].

#### 2.5.2. Correlation Analysis

Correlation analysis was used to determine the relationship between changes in PA behavior and biochemistry. Correlations were determined between PA behavior (LI values for the acquisition and consolidation of long-term memory) and the concentrations of oxidative stress biomarkers (TAC, SOD, and MDA) in the brain induced by acute administration of CB compounds.

For the correlation analysis StatSoft, Inc. (2011), STATISTICA (data analysis software system), version 10, http://www.statsoft.com/, was used. After performing the test for normality the Pearsonian Coefficient of Correlation was executed to determine the existence of the relationship between the given factors. The confidence limit of *p* < 0.05 was considered statistically significant.

## 3. Results

### 3.1. Results of Behavioral Experiments

#### 3.1.1. Influence of Selective or Nonselective CB Receptor Ligands on the Locomotor Activity in Mice

One-way ANOVA revealed that administration of the acute ip doses of CB receptor ligands had no statistically significant effect on the locomotor activity as compared with the appropriate control vehicle-injected groups (for CB2 receptor agonist, JWH 133: *F*(4,45) = 0.1459, *p* = 0.9639; for CB2 receptor antagonist, AM 630: *F*(5,54) = 1.720, *p* = 0.1458; for CB1/CB2 receptor agonist, WIN 55,212-2: *F*(3,36) = 1.138, *p* = 0.3468; and for CB1 receptor antagonist, AM 251: *F*(4,45) = 2.464, *p* = 0.0585) (Tables 1(a), 1(b), 1(c), and 1(d), resp.).

#### 3.1.2. The Influence of Selective or Nonselective CB Receptor Ligands on the Long-Term Acquisition and Consolidation of Memory and Learning Processes during Retention Trial in the PA Test

**Figure 1:**
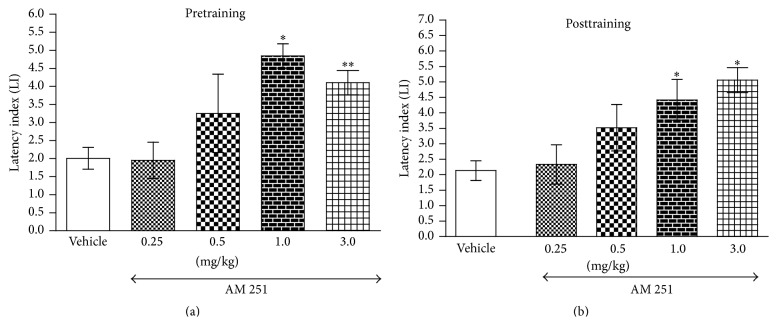
Effects of acute pretraining (a) or posttraining (b) CB1 receptor antagonist AM 251 or saline administration on the latency index (LI) in the PA test in mice. AM 251 (0.25, 0.5, 1.0, and 3.0 mg/kg; ip) or vehicle was administered 30 min before the first trial (a) or immediately after the first trial (b) and the mice were retested 24 h later; *n* = 8–12; the means ± SEM; ^*∗*^ *p* < 0.05; ^*∗∗*^ *p* < 0.01 versus vehicle-treated control group; Tukey's test.

One-way ANOVA revealed that pretraining administration of acute ip doses of an antagonist of CB1 receptors AM 251 (0.25, 0.5, 1.0, and 3.0 mg/kg) had a statistically significant effect on LI values (*F*(4,41) = 5.642; *p* = 0.0010). Indeed, treatment with AM 251 (1.0 and 3.0 mg/kg) significantly increased IL values in mice compared to those in vehicle-treated control group (*p* < 0.05 and *p* < 0.01, resp., post hoc Tukey's test) (Figure 1(a)), indicating that AM 251, at these used doses, improved the long-term acquisition of memory and learning. Similarly, Figure 1(b) shows that, for long-term memory consolidation during the retention trial, acute ip posttraining administration of AM 251 (0.25, 0.5, 1.0, and 3.0 mg/kg) significantly increased the LI values (*F*(4,35) = 5.190; *p* = 0.0022, one-way ANOVA) compared to vehicle-treated control mice. Furthermore, a post hoc Tukey's test revealed a statistically significant effect caused by treatment with 1.0 and 3.0 mg/kg of AM 251 (*p* < 0.05), which indicates that AM 251, at the used doses, also improved this stage of the memory and learning processes.

**Figure 2:**
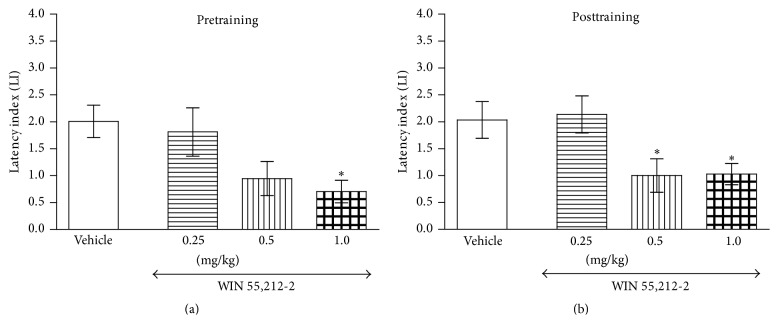
Effects of acute pretraining (a) or posttraining (b) CB1/CB2 receptor agonist WIN 55,212-2 or saline administration on the latency index (LI) in the PA test in mice. WIN 55,212-2 (0.25, 0.5, and 1.0 mg/kg; ip) or vehicle was administered 30 min before the first trial (a) or immediately after the first trial (b) and the mice were retested 24 h later; *n* = 8–12; the means ± SEM; ^*∗*^ *p* < 0.05 versus vehicle-treated control group; Tukey's test.

In turn, an acute ip pretraining and posttraining injection of CB1/CB2 receptor agonist WIN 55,212-2 (0.25, 0.5, and 1.0 mg/kg) significantly decreased LI values for long-term acquisition (*F*(3,35) = 3.687; *p* = 0.0209, one-way ANOVA) and consolidation trials (*F*(3,30) = 4.091; *p* = 0.0151, one-way ANOVA) as compared with vehicle-treated control mice. The post hoc Tukey's test confirmed a statistically significant effect: for memory acquisition during the retention trial (*p* < 0.05 for dose of 1.0 mg/kg) (Figure 2(a)) and for memory consolidation during the retention trial (*p* < 0.05 for doses of 0.5 and 1.0 mg/kg) (Figure 2(b)), indicating that WIN 55,212-2, at the used doses, impaired different stages of memory and learning processes.

**Figure 3:**
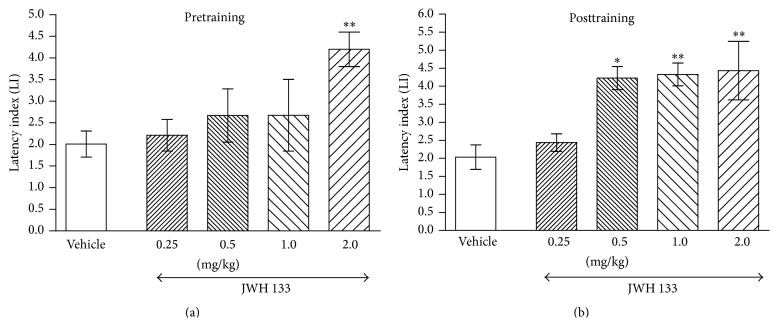
Effects of acute pretraining (a) or posttraining (b) CB2 receptor agonist JWH 133 or saline administration on the latency index (LI) in the PA test in mice. JWH 133 (0.25, 0.5, 1.0, and 2.0 mg/kg; ip) or vehicle was administered 30 min before the first trial (a) or immediately after the first trial (b) and the mice were retested 24 h later; *n* = 8–12; the means ± SEM; ^*∗*^ *p* < 0.05; ^*∗∗*^ *p* < 0.01 versus vehicle-treated control group; Tukey's test.

In the next experiments, one-way ANOVA revealed that the acute ip pretraining administration of CB2 receptors agonist JWH 133 (0.25, 0.5, 1.0, and 2.0 mg/kg) had a statistically significant effect on LI values (*F*(4,41) = 3.378; *p* = 0.0171) in the PA task. Indeed, the post hoc Tukey's test revealed that JWH 133, at the dose of 2.0 mg/kg, significantly increased LI values compared with vehicle-treated control mice, indicating that JWH 133 improved acquisition of the memory and learning processes (*p* < 0.01) (Figure 3(a)). Similarly, for long-term memory consolidation during the retention trial, posttraining injection of JWH 133 (0.25, 0.5, 1.0, and 2.0 mg/kg) had a statistically significant effect on LI values in the PA task compared to vehicle-treated control mice (*F*(4,32) = 7.065; *p* = 0.0003, one-way ANOVA). Furthermore, the post hoc Tukey's test confirmed a statistically significant effect (*p* < 0.05 for dose of 0.5 mg/kg and *p* < 0.01 for doses of 1.0 and 2.0 mg/kg) (Figure 3(b)), indicating that JWH 133, at the used doses, also improved this stage of memory and learning processes.

**Figure 4:**
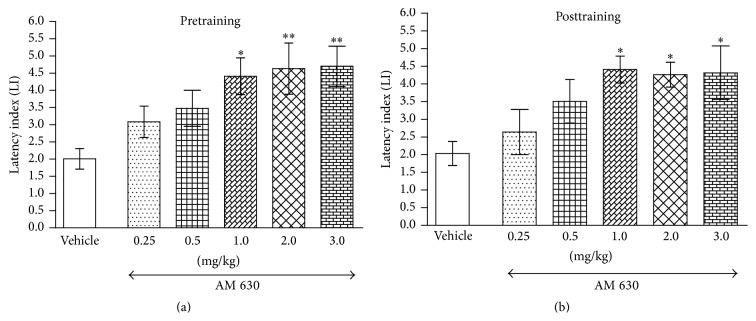
Effects of acute pretraining (a) or posttraining (b) CB2 receptor antagonist AM 630 or saline administration on the latency index (LI) in the PA test in mice. AM 630 (0.25, 0.5, 1.0, 2.0, and 3.0 mg/kg; ip) or vehicle was administered 30 min before the first trial (a) or immediately after the first trial (b) and the mice were retested 24 h later; *n* = 8–12; the means ± SEM; ^*∗*^ *p* < 0.05; ^*∗∗*^ *p* < 0.01 versus vehicle-treated control group; Tukey's test.

An interesting effect was observed when AM 630, an antagonist of CB2 receptors, was tested in the PA task. One-way ANOVA revealed that the acute ip pretraining administration of AM 630 (0.25, 0.5, 1.0, 2.0, and 3.0 mg/kg) caused a statistically significant change in LI values (*F*(5,47) = 5.552; *p* = 0.0004), with respect to long-term memory. The post hoc Tukey's test revealed a statistically significant improvement in memory and learning processes in animals that received acute doses of AM 630 (*p* < 0.05 for dose of 1.0 mg/kg and *p* < 0.01 for doses of 2.0 and 3.0 mg/kg) (Figure 4(a)). Similarly, for long-term memory consolidation during the retention trial, the mice receiving an acute ip posttraining injection of AM 630 (0.25, 0.5, 1.0, 2.0, and 3.0 mg/kg) had a statistically significant effect on LI values in the PA task compared to vehicle-treated control mice (*F*(5,41) = 3.459; *p* = 0.0107, one-way ANOVA). Additionally, the post hoc Tukey's test confirmed a statistically significant effect (*p* < 0.05 for doses of 1.0, 2.0, and 3.0 mg/kg) (Figure 4(b)), indicating that AM 630, at the used doses, also improved consolidation of memory and learning during retention trial.

### 3.2. Results of Biochemical Experiments

#### 3.2.1. Influence of Selective or Nonselective CB Receptor Ligands on the Level of Oxidative Stress Biomarkers in the Brain of Mice

Statistical analysis revealed that an acute administration of CB receptors ligands influenced antioxidant potential of brain tissue, expressed as increase in TAC values (one-way ANOVA (*F*(10,77) = 5.185; *p* < 0.0001)). The post hoc Tukey's test confirmed statistically significant increase in TAC value in brains of animals, which received a single dose of CB2 receptor ligands JWH 133 (*p* < 0.05 for dose of 2.0 mg/kg), AM 630 (*p* < 0.01 for dose of 2.0 mg/kg and *p* < 0.001 for dose of 3.0 mg/kg), and WIN 55,212-2 (*p* < 0.01 for dose of 1.0 mg/kg) in comparison to vehicle-treated control group (Table 2).

However, an acute administration of the used CB receptors ligands did not influence activity of SOD (one-way ANOVA analysis (*F*(10,92) = 1.302; *p* = 0.2411)) in statistically significant way. Indeed, Tukey's post hoc test did not show any statistically significant differences between cannabinoid compounds-treated groups and vehicle-treated control group (Table 2).

On the other hand, for the level of MDA, the main product of lipids peroxidation in brain, one-way ANOVA analyses revealed that an acute injection of CB receptors ligands had statistically significant changes in concentration of MDA (*F*(10,76) = 4.804; *p* < 0.0001). Indeed, the post hoc Tukey's test showed a statistically significant increased level of the MDA in examined brain, in the animals that acutely received JWH 133 (*p* < 0.05 for dose of 1.0 mg/kg), AM 630 (*p* < 0.05 for dose of 1.0), and AM 251 (*p* < 0.05 for doses of 1.0 and 3.0 mg/kg) as compared with vehicle-treated control group (Table 2).

We have determined the parameters of oxidative stress in brains of animals receiving all of the doses of CB receptor ligands used in the behavioral experiments; however, the results obtained for the lowest doses were not effective in the biochemical experiments versus vehicle-treated control group (data not shown).

### 3.3. Results of the Correlation Analysis

**Figure 5:**
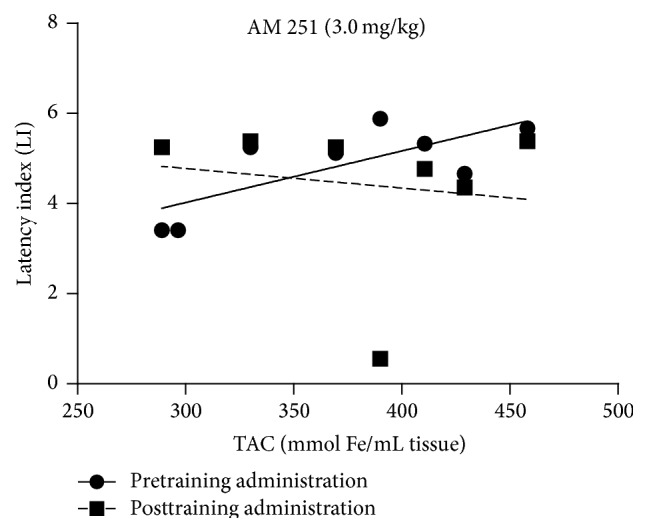
Correlations between the change in LI values in the PA test for the acquisition or consolidation of long-term memory and the concentration of TAC in the mice brain induced by the acute pretraining or posttraining injection of CB1 receptor antagonist AM 251 at the dose of 3.0 mg/kg.

For the relationship between the changes in the LI values in the PA test and the level of* TAC* in the brain, performed correlation analysis revealed existence of statistical significant correlation for pretraining administration of CB1 receptor agonist AM 251 at the dose of 3.0 mg/kg. LI values for the acquisition of long-term memory and the level of TAC in the brain tend to increase together (*r* ^2^ = 0.55; *p* = 0.035). However, no statistically significant correlation was received for posttraining administration of AM 251 (3.0 mg/kg) (*p* = 0.7571) (Figure 5).

**Figure 6:**
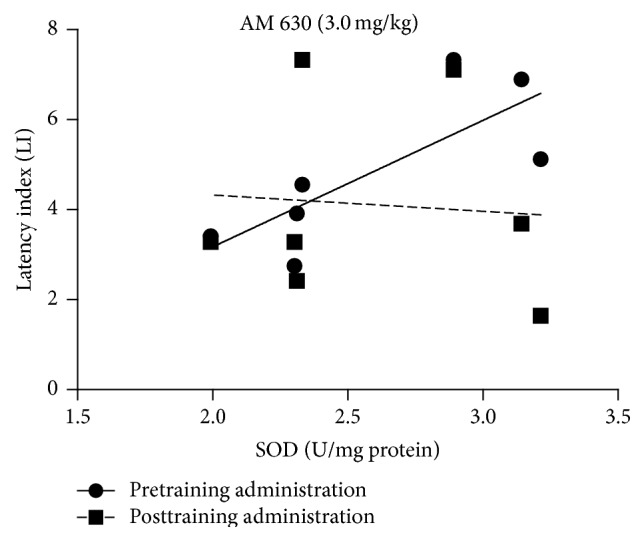
Correlations between the change in LI values in the PA test for the acquisition or consolidation of long-term memory and the concentration of SOD in the mice brain induced by the acute pretraining or posttraining injection of CB2 receptor antagonist AM 630 at the dose of 3.0 mg/kg.

For the relationship between changes in the LI values in the PA test and the level of* SOD* in the brain, performed correlation analysis showed a strong statistically significant correlation for pretraining injection of CB2 receptor agonist AM 630 at the dose of 3.0 mg/kg. LI values for the acquisition of long-term memory and the level of the SOD in the brain tend to increase together (*r* ^2^ = 0.61; *p* = 0.039). However, there was no statistical correlation between LI values for posttraining injection of AM 630 (3.0 mg/kg) and the level of SOD (*p* = 0.868) (Figure 6).

**Figure 7:**
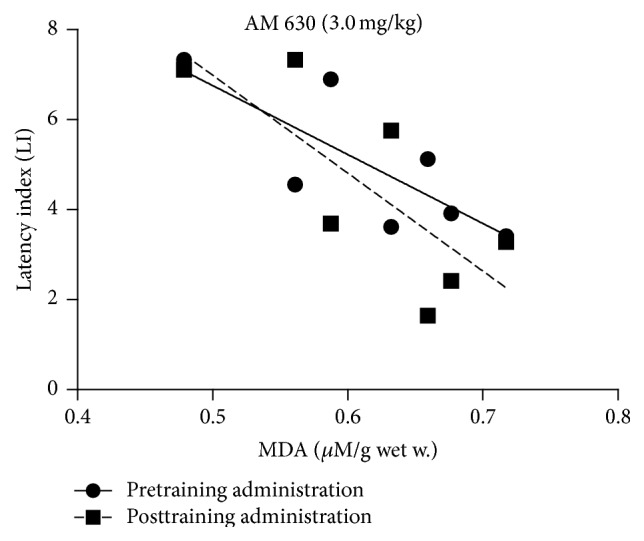
Correlations between the change in LI values in the PA test for the acquisition or consolidation of long-term memory and the concentration of MDA in the mice brain induced by the acute pretraining or posttraining injection of CB2 receptor antagonist AM 630 at the dose of 3.0 mg/kg.

For the relationship between changes in the LI values in the PA test and the level of* MDA* in the brain, the statistically significant strong correlation was found for the systemic pretraining and posttraining administration of CB2 receptor agonist AM 630 at the dose of 3.0 mg/kg. With the decrease of LI values for the acquisition of long-term memory the level of MDA in the brain increased (*r* ^2^ = 0.6122; *p* = 0.0376) as well as for the consolidation of long-term memory (*r* ^2^ = 0.5909; *p* = 0.0434) (Figure 7).

No more statistically significant correlations were found between all tested factors (data not shown).

## 4. Discussion

The aim of the present experiments was to examine the involvement of the endocannabinoid system through CB1 as well as CB2 receptors in the different stages of memory in the PA test in Swiss male mice. Moreover, for the first time to our knowledge, we evaluated the influence of selective or nonselective CB ligands on the level of oxidative stress in the whole brain in mice and assessed all possible correlations between behavioral and biochemical effects.

Our results have shown that, in the PA test, a single pretraining and posttraining administration of a selective CB1 antagonist, AM 251 (1.0–3.0 mg/kg), significantly improved long-term memory processes, although AM 251 caused the increase in the MDA level in the brain. Nevertheless, correlation analysis revealed that this improvement of long-term memory acquisition induced by an acute pretraining injection of AM 251, at the dose of 3.0 mg/kg, was strongly correlated with the increased TAC level in the brain. What is interesting is that we revealed that an acute systemic pretraining or posttraining administration of a mixed CB1/CB2 receptor agonist, WIN 55,212-2, at the dose of 1.0 mg/kg, impaired cognitive processes in the PA test but at the same time increased the level of TAC in the brain showing certain antioxidant properties. However, to strictly compare the involvement of CB1 receptors in the memory-related responses, further research with the use of selective CB1 receptor agonist is needed.

In the next step of our experiments, we demonstrated that an acute pretraining or posttraining administration of a selective CB2 receptor agonist JWH 133 and a CB2 receptor antagonist AM 630 significantly improved memory-related responses in the PA test in mice and exhibited the antioxidant properties in dose-dependent manner observed as the increase in level of TAC in the brain. The lower dose of JWH 133 (0.5 mg/kg) did not change the level of antioxidant barrier parameters and had no influence on the acquisition of long-term memory but enhanced the consolidation of long-term memory in the PA test. The higher doses of JWH 133 improved acquisition or consolidation of long-term memory (for doses of 1.0 and 2.0 mg/kg) and exhibited antioxidant effect, increasing TAC level in the brain (for the dose of 2.0 mg/kg).

In turn, for the lower dose of AM 630 (0.5 mg/kg), a CB2 receptor antagonist was found inactive; that is, it did not affect antioxidant barrier parameters and did not change memory-related responses in the PA test in mice. The higher doses of AM 630 (2.0 and 3.0 mg/kg) induced statistically significant increase in antioxidant property of brain tissue and caused long-term memory improvement in behavioral test. Additionally, it should be noted that according to our results the impact of AM 630 on the level of oxidative stress biomarkers in mice brain seems to be strongly correlated with the improvement of memory in the PA in mice. Correlation analysis showed that this enhancement of long-term memory acquisition induced by an acute pretraining administration of AM 630 at the dose of 3.0 mg/kg was correlated with the decreased TAC level in the brain. Furthermore, for the relationship between improvement of long-term memory acquisition in the PA test in mice and the level of SOD in the brain, performed correlation analysis demonstrated a strong statistically significant correlation for an acute pretraining injection of AM 630 at the dose of 3.0 mg/kg, although in the biochemical study none of the CB compounds influenced the activity of SOD.

Presented studies are our preliminary studies as we were curious if a single injection of antioxidant substances can affect oxidative balance within the whole brain at all. As the experiment gives us positive feedback, further research is required to test whether CB ligands modulate the oxidative stress level in areas strictly related to memory processing, for example, the frontal cortex and the dorsal hippocampus.

The influence of the CB1 receptor ligands on memory and learning processes has been widely documented by various experiments and clinical studies [38–40].

Animal studies have demonstrated that an acute administration of CB1 agonists (e.g., natural agonist, Δ9-THC, and synthetic agonists, CP55940 and HU-210) and also pretraining administration of CB1/CB2 mixed agonist, WIN 55,212-2, attenuated acquisition of memory in various animal models, for example, object recognition task, water maze test, and contextual fear conditioning test [41–44]. Similarly, chronic administration of WIN 55,212-2 significantly impaired spatial memory in rats evaluated in the water maze test [27]. Furthermore, indirect stimulation of CB1 receptors impaired acquisition of memory in the recognition memory test [45]. Additionally, it has been revealed that activation of CB1 receptors in the BLA potentiated fear memory acquisition. In turn, inhibition of CB1 receptors in the BLA blocked the acquisition of olfactory fear memory [46]. On the other hand, an acute pretraining administration of the CB1 antagonist, SR-141716A, facilitated acquisition of memory in rodents observed in the PA test, elevated T-maze test, and social recognition memory task [47, 48] or impaired acquisition of memory assessed in the spatial memory test [49].

Contradictory data concerning the influence of CB1 on the consolidation of memory have been also reported. It has been demonstrated that posttraining administration of CB1 receptor agonist (HU-210), a mixed CB1/CB2 receptor agonist (WIN 55,212-2), or indirect CB1 receptor agonist (URB597) attenuated consolidation of memory in the contextual fear conditioning, water maze test, and object recognition test [50–53]. However, intra-BLA infusion of WIN55,212-2 facilitated memory consolidation in rats evaluated in the inhibitory avoidance task or had no effect in mentioned animal model [54, 55]. In turn, posttraining intrahippocampal injection of this drug impaired consolidation of memory in several behavioral tasks [56, 57]. On the other hand, systemic posttraining administration of CB1 receptor antagonist, rimonabant, enhanced memory consolidation in the radial-arm maze test, elevated T-maze test, or eight-arm radial maze task [48, 58, 59]. Interestingly, posttraining injection of another CB1 antagonist, AM 251, interfered in consolidation of memory-related processes in the step-through inhibitory avoidance task or contextual fear conditioning [54, 60].

Such contradictory findings reported and our results may be connected with differences in behavioral tasks used, handling procedures, for example, time of drug administration, the kind of drug treatment, or other experimental conditions, as well as doses and CB compounds selected. Moreover, it should be assessed whether CB compounds affect cognition per se or by other nonspecific mechanisms. The limitations mentioned below will be discussed in this section.

The first limitation is connected with the time of drug administration. The interpretation of the results from studies concerning the pretraining and posttraining drug administration is very difficult because such treatments may have influence on diverse processes. For example, after pretraining administration of cannabinoids, these compounds may strongly alter pain perception and locomotor activity at the time of training, thus adding significant potential confounds occurring when a drug is given before the training. Therefore, it is difficult to discriminate between the influence only on the cognitive effects and confounding variables (e.g., alteration in locomotor activity [61], pain sensitivity [62], and/or motivation) following pretraining CB compounds administration [39]. Thus, drugs can be administered after a training event to isolate the phase of memory consolidation and exclude influences on acquisition or any motor or motivational processes that may have impact on the learning indirectly. Additionally, since CB compounds alter the motor activity and may give false positive and negative effects in other behavioral tests, an additional test should be carried out with the specific aim of monitoring locomotor activity. For this purpose, we evaluated the influence of an acute administration of CB receptor ligands on the horizontal locomotion in mice. Our results showed that none of the ligands used had any influence on the locomotion of mice, confirming the results obtained in our previous experiments [26]. Therefore, by measuring locomotor activity, for the following experiments focused on the memory-related behavior, we have used only these doses of CB compounds that did not change the locomotor activity of mice, suggesting that it is very unlikely that the observed cognitive-related effects induced by CB receptor ligands are false positives or negatives.

Moreover, conflicting data have been reported concerning the effects on memory performance of infusing CB drugs locally into discrete brain regions. As we mentioned previously, CB1 receptors are highly expressed in brain structures that are critical for emotional- and cognition-related processes, including the BLA, the mPFC, and the hippocampus. Due to their localization, CB1 receptors are critically involved in the control of consolidation and extinction of emotionally salient events within the amygdala-prefrontal cortical pathways [46, 54, 63]. However, in our experiments we did not administer CB receptor ligands directly into the particular brain regions; therefore, results presented in our paper concerning the influence of CB receptor ligands on the acquisition or consolidation of memory are due to their systemic administration.

Additionally, several findings suggest that endocannabinoid system, through the CB (mainly CB1) receptors, is involved in the modulation of the anxiety and fear-related behaviors. There is a general consensus that the effects of CB1 receptor agonists on anxiety seem to be biphasic with low doses being anxiolytic and high doses possibly anxiogenic [64, 65]. The main problem with the lack of convergence of the data may lie in the lack of selectivity of the CB1 receptor ligands, the possible inverse agonistic properties of most CB1 receptor antagonists, and the involvement of different CB1 and non-CB1 receptor subtypes in the behavioral effects. Additionally, CB2 receptor agonists and antagonists may provoke anxiolytic or anxiogenic effects, depending on the acute or chronic administration [24]. Therefore, taking into account the above presented literature data, we cannot exclude the influence of the anxiety levels on the memory in the PA task, and more detailed knowledge of these limitations needs further investigations.

Another limitation that may have influence on the memory-related responses provoked by cannabinoids is connected with the selectivity of CB compounds used and their mechanisms of action. Concerning possible neuronal mechanisms of biochemical and behavioral effects revealed in our study, it is worth mentioning that the activation of CB1 receptors inhibits neurotransmitter release by modulating several ion channels (e.g., voltage-gated calcium channels, potassium channels) and kinases [66, 67]. These processes suppress calcium and activate inward-rectifying potassium conductance effects associated with depression of neuronal excitability and transmitter release. Additionally, CB1 receptors play a key role in modulation of synaptic transmissions, for example, glutamatergic, serotonergic, noradrenergic, cholinergic, and dopaminergic [46, 68–71]. It has been shown that the blockade of CB1 receptors increases release of many neurotransmitters (including ACh, neurotransmitter essential for memory and learning processes), thus improving cognitive processes [65]. On the other hand, activation of CB1 receptors inhibits gamma-aminobutyric acid- (GABA-) related neurotransmission in the hippocampus and thus may attenuate formation of memory pathways [69, 72].

The improvement of memory caused by CB1 receptor ligands presented in our paper was obtained rather through their receptor mediated action; however, the specific impact of CB2 receptor ligands on the cognition-related processes seems to be more complex and yet not precisely explored. In our behavioral studies, we reveled that both a selective CB2 receptor agonist JWH 133 and a competitive CB2 receptor antagonist AM 630 significantly improved long-term memory acquisition and consolidation in the PA test. However, in contrast to our findings, García-Gutiérrez et al. [25] have shown that JWH 133 enhances memory consolidation but AM 630 impairs it in the step-down inhibitory avoidance test.

The enhancement of memory caused by both CB2 antagonist and CB2 agonist obtained in our studies may be connected with pharmacokinetic properties of tested CB2 receptor ligands, that is, JWH 133 and AM 630. However, the CB2 selective agent, AM 630, behaves as inverse agonist rather than as “silent” antagonist. Not only the inverse efficacy at CB2 receptors but also the CB2/CB1 affinity ratio has been indicated for AM 630 (CB2/CB1 affinity = 165). Thus, AM 630 has been found to behave as a low-affinity partial agonist in some experiments but as a low-potency inverse agonist in another study [67]. The pharmacological properties of AM 630 are more complex. It has been revealed that AM 630 behaves as an inverse agonist at CB2 receptors as well as an inverse agonist at CB1 receptors [73, 74]. Thus, we may propose that both agonist and antagonist of CB2 receptors used in our study may improve memory and learning processes through CB1 as well as CB2 receptors. However, further experiments are required to explain this phenomenon.

Moreover, in our research, the improvement of memory induced by CB2 receptor ligands is probably associated with antioxidant properties, exhibited by both agonists and antagonist of these receptors [75].

We found out that CB2 receptor ligands significantly improved antioxidant properties of brain tissue in dose-dependent manner, while increase in TAC value observed in case of CB1 receptor ligands was rather slight. Moreover, changes in MDA concentration in the brain confirmed antioxidant effect of CB2 receptor ligands, while the level of MDA after administration of CB1 receptor compounds was significantly increased, indicating the intensification of lipids peroxidation processes.

In general, the drugs that improve learning and memory in animals at the same time significantly reduced the level of MDA in the brain [10–13]. However, in our experiments we observed that AM 251, a CB1 receptor antagonist, improved cognitive-related processes assessed in the PA test and caused the increase in concentration of MDA. Additionally, we have also performed studies on mephedrone, a synthetic club drug, that induced oxidative stress within brain and its structures responsible for the cognitive functions and also facilitated acquisition and consolidation of the memory processes at the same time [76]. Similar relationship was observed in the study dealing with nicotine, a nicotinic receptor agonist, which is also prooxidative drug that improves memory functions [34, 76].

Based on the cited data, we can suspect that the mechanisms of cognitive function improvement were more dependent on receptors action of the drugs rather than on their prooxidative properties. In particular, the improvement of memory caused by CB1 receptor ligands presented in our paper was obtained rather through their receptor mediated action.

These procognitive effects provoked by CB receptor ligands may come from proper CB receptors expression in particular tissues. As CB1 receptors are mainly expressed in neurons and glial cells, they may regulate numerous brain functions, like cognition, emotion, motor control, feeding, and pain perception, in receptor-dependent manner [77, 78]. CB2 receptors, instead, are mainly localized in cells of immune system and their expression in some neurons has been found relatively low. Therefore, we hypothesize their memory improving action through nonreceptor mediated but rather direct antioxidant effect on neurons in brain tissue.

Moreover, regarding increased process of lipids peroxidation expressed by increase in MDA concentration, the CNS is very susceptible to oxidative stress as the brain has a high consumption of oxygen, contains large amounts of free-radical generating iron and substances like ascorbate, glutamate, and polyunsaturated fatty acids, which easily undergo redox-reaction leading to radicals' formation, and exhibits relatively poor antioxidant defense systems. Therefore, lipid peroxidation processes are very common within the brain and may be inhibited or accelerated by applied exogenous substances [3].

Additionally, in our experiment none of applied ligands had any effect on activity of SOD, an enzyme that plays a key role in neuronal protection against the damaging effects of superoxide anions in brain tissue. One possible hypothesis may be their direct free radicals scavenging properties, which results from their chemical structure, although we cannot exclude possible modulation of signaling pathways as an important mode of action likely responsible of the neuroprotective effect of CB compounds.

Furthermore, although they do not possess phenolic moieties, numerous unsaturated bonds and their lipophilic character make them able to scavenge reactive radicals similarly to low-molecular weight antioxidant molecules as glutathione (GSH), tocopherols, ascorbic acid, or exogenous flavonoids in brain tissue. Brain is quite vulnerable to ROS-mediated oxidative damages due to high concentration of polyunsaturated fatty acids, high consumption of oxygen, and large amounts of free-radical generating iron and other substances [79]. Antioxidant neuroprotective properties of phenolic and nonphenolic CB compounds and the involvement of CB1 in these effects were analyzed in detail using in vitro models of oxidative stress and neurodegeneration [17]. The study reveals that CB1 receptor is not directly involved in the mechanism in which antioxidant cannabinoids protect neuronal cells against oxidative stress. The authors postulate CB1 receptor-mediated and direct antioxidant action of phenolic cannabinoids and only receptor-dependent manner for nonphenolic ones. Our research showed strong antioxidant effect of WIN 55,212-2, although it does not contain phenolic moiety. In the study concerning endocannabinoid system involvement in regulating oxidized low density lipoprotein- (oxLDL-) induced inflammation and oxidative stress in macrophages, WIN 55,212-2 reduces production of ROS mainly via activation of CB1/CB2 receptor signaling [80].

Interestingly, it has been reported that also through chemical structure of CB2 receptors CB2 receptor ligands possess antioxidant effect, that is, scavenging reactive oxygen species and therefore reduction in oxidative stress and neuroprotection [75]. Furthermore, Walter et al. [20] have found that the level of expression of CB2 (and also CB1) receptors or concentrations of endocannabinoids in the brain are dramatically enhanced in time in the specific parts of the brain (e.g., glial cells and microglial) during the neurodegenerative processes [29, 81, 82].

Additionally, it should be noted that activation of CB2 receptors inhibits adenylate cyclase [83, 84] and activates mitogen-activated protein kinase [83, 85] through the G protein as in the case of CB1 receptors. However, in contrast to CB1 receptors, effects of CB2 selective receptor ligands are not connected with ion channels (e.g., calcium or potassium) and therefore they show lack of side effects from the CNS that occur during the use of CB1 receptor ligands [78]. Therefore, the use of CB2 receptor ligands to inhibit oxidative stress damages associated with memory impairment seems to be safer than use of CB1 receptor ligands.

## 5. Conclusion

Our results indicate a pharmacological approach for a role of the endocannabinoid system (through both CB1 and CB2 receptors) in the different stages of long-term memory and in the level of the oxidative stress-related parameters in the whole brain in mice. CB compounds combine both memory-related improvement ability and antioxidant properties.

Therefore, the use of CB receptor ligands (especially CB2 receptor ligands) to inhibit oxidative stress damages associated with memory impairment may be important for the treatment of many of cognitive dysfunctions. However, more detailed knowledge of the involvement of endocannabinoid system in the processes and brain areas connected with memory and learning as well as the influence of CB receptor ligands on the memory impairment observed in the animal models, for example, animal models of AD, deserves further investigation.

## Figures and Tables

**(a):** 

Drugs	Photocell beam breaks ± SEM (60 min)
Vehicle	588.9 ± 61.20
JWH 133 (0.25 mg/kg)	611.7 ± 25.11
JWH 133 (0.5 mg/kg)	644.2 ± 74.15
JWH 133 (1.0 mg/kg)	606.5 ± 45.15
JWH 133 (2.0 mg/kg)	621.3 ± 47.77

**(b):** 

Drugs	Photocell beam breaks ± SEM (60 min)
Vehicle	597.0 ± 29.63
AM 630 (0.25 mg/kg)	646.25 ± 26.45
AM 630 (0.5 mg/kg)	677.84 ± 34.69
AM 630 (1.0 mg/kg)	743.01 ± 42.86
AM 630 (2.0 mg/kg)	724.44 ± 22.05
AM 630 (3.0 mg/kg)	712.9 ± 73.16

**(c):** 

Drugs	Photocell beam breaks ± SEM (60 min)
Vehicle	588.9 ± 61.20
WIN 55,212-2 (0.25 mg/kg)	490.5 ± 77.03
WIN 55,212-2 (0.5 mg/kg)	422.6 ± 87.20
WIN 55,212-2 (1.0 mg/kg)	414.8 ± 74.71

**(d):** 

Drugs	Photocell beam breaks ± SEM (60 min)
Vehicle	555.15 ± 48.12
AM 251 (0.25 mg/kg)	406.0 ± 60.29
AM 251 (0.5 mg/kg)	394.71 ± 24.43
AM 251 (1.0 mg/kg)	445.54 ± 42.07
AM 251 (3.0 mg/kg)	517.5 ± 40.66

**Table 2:** Effect of an acute administration of cannabinoid receptor ligands on oxidative stress biomarkers in the whole brains of mice. Data are presented as the means ± SEM; *n* = 8–12; ^*∗*^ *p* < 0.05, ^*∗∗*^ *p* < 0.01, and  ^*∗∗∗*^ *p* < 0.001 versus vehicle-treated control group; Tukey's test.

Drug (dose)	TAC	SOD	MDA
	[mmol Fe/mL tissue]	[U/mg protein]	[*μ*M/g wet w.]
Vehicle	249.3 ± 28.54	2.458 ± 0.1279	0.596 ± 0.027
JWH 133 (0.5 mg/kg)	275.7 ± 21.79	2.516 ± 0.1131	0.653 ± 0.029
JWH 133 (1.0 mg/kg)	385.1 ± 32.20	2.894 ± 0.1422	0.915 ± 0.056^*∗*^
JWH 133 (2.0 mg/kg)	404.0 ± 31.68^*∗*^	2.648 ± 0.1071	0.817 ± 0.080
AM 630 (0.5 mg/kg)	271.7 ± 19.89	2.349 ± 0.1267	0.611 ± 0.047
AM 630 (1.0 mg/kg)	327.4 ± 30.87	2.367 ± 0.1219	0.905 ± 0.094^*∗*^
AM 630 (2.0 mg/kg)	401.0 ± 32.44^*∗∗*^	2.505 ± 0.1371	0.880 ± 0.065
AM 630 (3.0 mg/kg)	446.4 ± 35.02^*∗∗∗*^	2.532 ± 0.1475	0.616 ± 0.030
WIN 55,212-2 (1.0 mg/kg)	418.8 ± 33.27^*∗∗*^	2.610 ± 0.1434	0.850 ± 0.059
AM 251 (1.0 mg/kg)	343.3 ± 25.83	2.437 ± 0.1258	0.900 ± 0.063^*∗*^
AM 251 (3.0 mg/kg)	371.5 ± 21.88	2.513 ± 0.1773	0.897 ± 0.078^*∗*^

## Abbreviations

ACh: Acetylcholine
AD: Alzheimer's disease
BLA: Basolateral amygdala
CB: Cannabinoid
CNS: Central nervous system
Fe(II)-TPTZ: Fe(II)-tripyridyltriazine
Fe(III)-TPTZ: Fe(III)-tripyridyltriazine
FRAP: Ferric reducing ability of plasma
GABA: Gamma-aminobutyric acid
GPx: Glutathione peroxidase
GSH: Glutathione
IL-1*β*: Interleukin-1*β*
LI: Latency index
mPFC: Medial prefrontal cortex
MDA: Malondialdehyde
oxLDL: Oxidized low density lipoprotein
PA: Passive avoidance
PFC: Medial prefrontal cortex
ROS: Reactive oxygen species
SOD: Superoxide dismutase
TAC: Total antioxidant capacity
TBA: Thiobarbituric acid
TL: Transfer latency
TNF-*α*: Tumor necrosis factor-*α*
XOD: Xanthine oxidase.
